# Randomized non-inferiority TrIal comParing reverse T And Protrusion versus double-kissing and crush Stenting for the treatment of complex left main bifurcation lesions

**DOI:** 10.1007/s00392-021-01972-2

**Published:** 2021-11-24

**Authors:** Maximilian Olschewski, Helen Ullrich, Maike Knorr, Giulio Makmur, Majid Ahoopai, Thomas Münzel, Tommaso Gori

**Affiliations:** 1 Department of Cardiology, University Medical Center Mainz, Langenbeckstrasse 1, 55131 Mainz, Germany; 2 German Center for Cardiac and Vascular Research (DZHK), Standort Rhein-Main, Germany

**Keywords:** Coronary bifurcation lesions, Percutaneous coronary interventions, Optical coherence tomography

## Abstract

**Background:**

The treatment of left main bifurcation stenoses remains challenging.

**Aims:**

We compare the “Reverse T and Protrusion” (reverse-TAP) technique to Double-Kissing and crush (DK-crush).

**Methods:**

The study was designed as non-inferiority trial, the primary endpoint was percentage stent expansion in the ostial side branch at optical coherence tomography.

**Results:**

52 consecutive patients (13 females, 17 diabetics, Syntax score 25 [22–29]) with complex coronary bifurcation lesions of the left main were randomized in a 1:1 ratio to Reverse-TAP or DK-crush stenting. The intervention was performed according to protocol in all patients in both randomization groups. Side branch stent expansion was 75 [67–90]% in the DK-crush group and 86 [75–95]% in the reverse-TAP group (one-sided 97.5% lower parametric confidence interval: − 0.28%; *P* < 0.01 for non-inferiority; *P* = 0.037 for superiority). Side branch balloon pressure during final kissing was higher in the DK-crush group (14 [12–16] vs. reverse-TAP: 13 [12–14]; *P* = 0.043). Procedural time was shorter with reverse-TAP (DK-crush: 32 [24–44] min vs reverse–TAP: 25 [22–33] min; *P* = 0.044). Other procedural parameters were not different between groups. There was no difference in any of the safety endpoints up to 1 month.

**Conclusions:**

A reverse-TAP strategy for the interventional treatment of complex coronary lesions was non-inferior and superior to DK-crush for the primary endpoint side branch expansion while requiring less time. A larger study testing long-term clinical outcomes is warranted.

**Trail Registration:**

NCT: NCT03714750.

**Graphical abstract:**

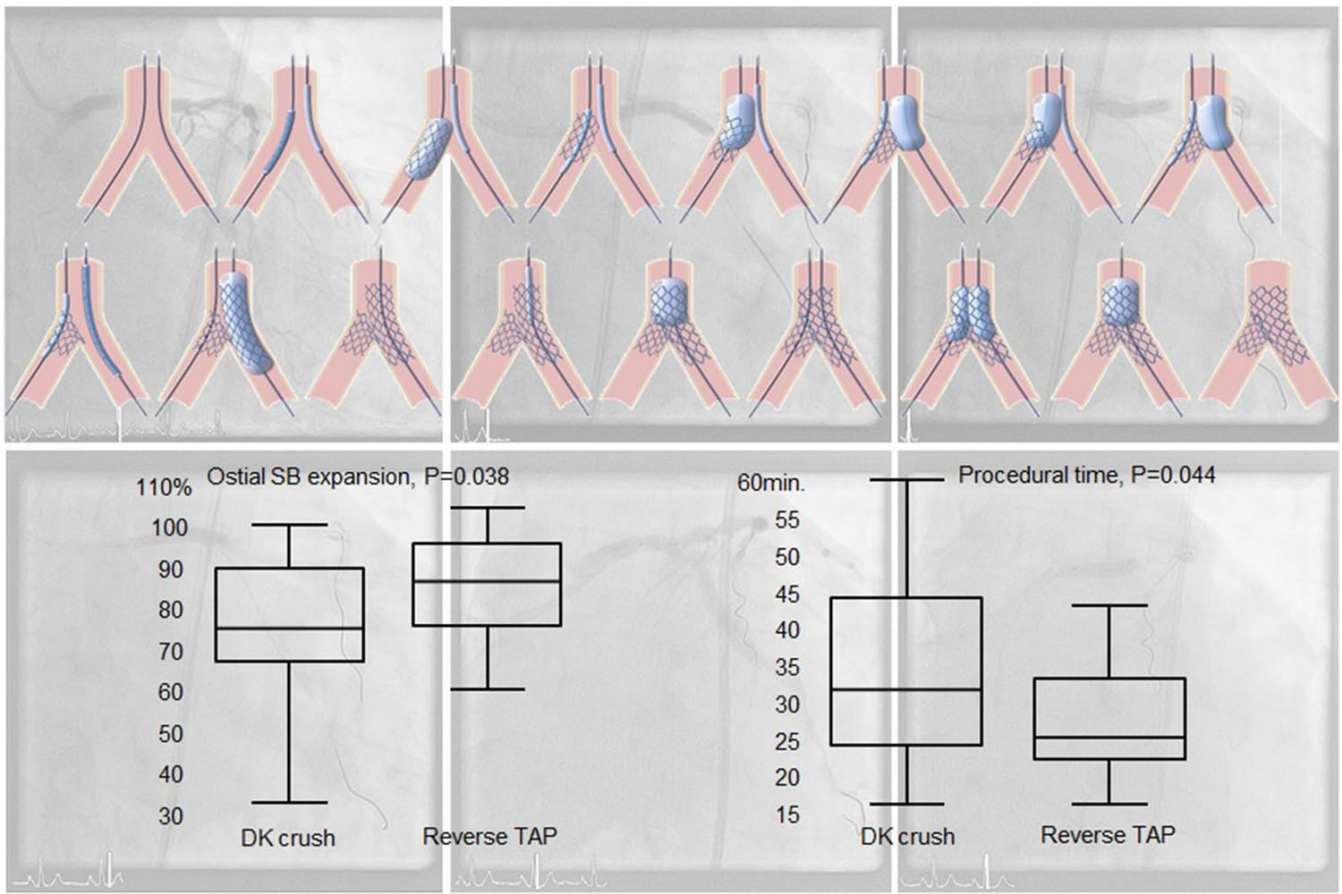

**Supplementary Information:**

The online version contains supplementary material available at 10.1007/s00392-021-01972-2.

## Introduction

Due to the large size of potentially jeopardized myocardium, unprotected left main coronary artery disease remains a clinical challenge. Percutaneous intervention is an alternative to by-pass surgery for selected patients [1], but these interventions require expertise and an adequate stenting strategy. In particular, controversy exists whether complex lesions (as defined by a set of major and minor criteria including calcification, lesion length, bifurcation angle, and diameter stenosis) should be treated with a planned 2-stent techniques rather than a simpler single-stenting provisional PCI. In the recent European Bifurcation Club paper, the incidence of events was actually numerically lower in the provisional layered approach as compared to planned 2-stent strategies [2]. Nonetheless, a 2-stent strategy may remain necessary in selected cases, and a lesion/patient-specific approach is necessary [3]. Among the different 2-stent techniques (summarized in the MADS classification and its amendments [4]), the double-kissing and crush (DK-crush) has been shown to result in improved stent expansion at the side branch (SB) ostium, and in reduced neointimal proliferation during 8-month follow-up [5]. DK-crush was associated with lower rates of target-lesion revascularization as compared with provisional or culotte stenting in both non-left main and left main coronary bifurcation lesions [6–9]. As compared to older techniques, DK-crush requires an additional kissing balloon dilation prior to main branch (MB) stenting. This kissing PTCA facilitates the second rewiring of the SB and the final kissing dilation but obviously implies an extra step.

To simplify the procedure and/or further improve outcomes, several technical refinements to the original DK-crush strategy have been proposed. Among others, an immediate high-pressure post-dilation of the SB stent, double-kissing culotte, “nanoˮ crush techniques, or the use of thin-strut stents which might help reduce strut volume and avoid malapposition in the overlap region [10, 11]. In the present study, we compared the DK-crush with a modified version of the so-called T-and-protrusion strategy requiring one single rewiring step. This planned 2-stent T-and-protrusion (“reverse-TAPˮ) technique was designed to simplify the procedure (thus reducing procedural time) by introducing two main modifications:

1. Alternate high-pressure dilations of MB and SB balloons following SB stent implantation and substituting the first kissing balloon. The rationale for this is that this step would allow a circumferential distribution of the SB stent struts instead of crushing on one single side.
2. No need for the first rewiring (and first kissing), substituted by the above alternate dilations.

Since SB restenosis remains the most frequent cause of failure after bifurcation stenting [12], we tested the hypothesis that reverse-TAP would be non-inferior to DK-crush in terms of ostial SB expansion while reducing procedural time.

## Methods

### Study design

The design of the “Randomized, non-inferiority, controlled procedural outcomes TrIal comParing reverse T And Protrusion versus double-kissing and crush Stenting” trial, as approved by the local ethics committee and published in clinicaltrials.org (NCT03714750) has been previously described [13]. The trial was funded by support from the German Ministry for Research and Education through the Deutsches Zentrum für Herz und Kreislauf Forschung, which otherwise did not participate in the design or conduct of the study, analysis, or interpretation of the data, or the decision to submit the manuscript for publication.

### Patient population

Consecutive patients with complex left main lesions and a heart team recommendation for interventional treatment of a Medina 1,1,1 or 0,1,1 left main lesion were evaluated for enrollment in the trial. Inclusion and exclusion criteria are listed in detail in the supplement. All patients provided written informed consent. Participating primary operators had performed 300 PCIs/year for at least 5 years, at least 20 left main PCIs/year with either technique.

**Fig. 1:**
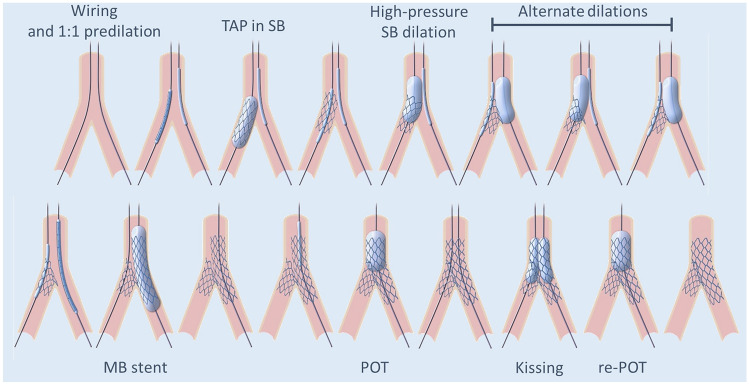
The reverse-TAP technique. Description and comparison with DK-crush in Table 1. The last proximal optimization technique (POT) dilation was not compulsory in either study arm

Patients were randomly assigned in a 1:1 ratio to either DK-crush or reverse-TAP blocks (each block 10 exams) without stratification. Randomization was based on a computer-generated random sequence (Medcalc, Mariakerke, Belgium) and was performed after wiring both main branch (MB) and SB. Procedural anticoagulation was achieved with unfractionated heparin and adjusted to maintain the activated clotting time > 250. Use of debulking devices and glycoprotein IIb/IIIa inhibitors was left to the operator’s discretion. The two PCI techniques have been previously described [13] and the main differences are summarized in Table 1 and Fig. 1. Stents diameters were chosen based on the reference diameter of the MB/SB segment distal to the bifurcation with an ideal stent/artery ratio slightly above 1:1. After completion of the PCI, optical coherence tomography (OCT) imaging was performed in all patients to collect the pre-defined study endpoints. Further optimization (post-dilation and/or stenting) based on current guidelines (latest updated in [14]) was left to the operator´s discretion and recorded. Patients were treated with aspirin pre-procedure and a P2Y12 inhibitor periprocedurally (clopidogrel for stable angina patients, ticagrelor, or prasugrel for acute coronary syndromes). After intervention, patients were prescribed guideline-directed dual antiplatelet therapy. Only Xience ProA stents (Abbott vascular, S.ta Clara, USA) were used.

**Table 1:** Differences between DK-crush and reverse-TAP

DK-crush [5]	Reverse-TAP
Vessel wiring and predilation	Vessel wiring and predilation
“Sentinelˮ balloon in the LM-MB	“Sentinelˮ balloon in the LM-MB
SB stenting with ~ 2 mm protrusion	**SB stenting with minimal protrusion**
The SB balloon and wire are removed	**The SB stent balloon is retracted half-way into the left main and inflated at 4–6 ATM above nominal pressure**
Crush of the SB stent	Sequential high-pressure inflations (2–3 each) followed by kissing*
SB rewiring and kissing	
MB stenting	MB stenting
POT	POT
Second SB rewiring through a non-distal cell and kissing	Rewiring through a distal cell and kissing
Final POT	Final POT

*POT* proximal optimization technique, *MB* main branch, *SB* side branch, *LM* left main

*Sequential balloon inflation (alternate inflation of MB and SB balloon followed by kissing) is designed to minimize stent distortion and SB dissection risk, and optimize expansion of the ostial SB and MB avoiding a three-layer crush in the left main and at the same time minimizing the longitudinal length of the neocarina. The differences between the two techniques are in bold characters. Note: slightly different descriptions of the DK-crush technique have been presented over time (SB rewiring through proximal vs distal cells, re-crush after first kissing, introduction of POT [3])

The description presented here is the one used in the current study. In both techniques, the ballons and stents were sized 1.0–1.1 to the distal vessels (MB/SB). POT balloons were sized to the left main

### Endpoints

#### Optical coherence tomography

OCT endpoints were collected after final kissing/proximal optimization using the Dragonfly™ Duo ILUMIEN™ (St. Jude Medical, St. Paul, MN, USA). Data were analyzed off-line using QCU-CMS Version 4-69 (Leiden University Medical Center and MEDIS, Leiden, Netherland) by staff blinded to the allocation group. In the segment comprised between the carina and anticarina, all analyses were carried out on a frame-by-frame basis to collect data on malapposition, SB opening, and MB/SB ostium expansion. Analyses were performed at 1-mm intervals in the left main proximal to the bifurcation, in the MB and SB to record mean, maximum and minimum stent diameter and cross-sectional area, stent asymmetry and eccentricity, and malapposition. Side branch opening was calculated as the mean and minimum % Ratio between the longest strut-free segment and the estimated ostium diameter [15] (Supplemental Fig. 1). The strut malapposition threshold was set at 110microm and 250microm. The primary endpoint was ostial side branch expansion, calculated as % ratio of stent area at SB ostium divided by the reference area [16]. The SB ostium was the first frame distal to the carina. As additional exploratory endpoint, the ratio of minimum ostial SB diameter to reference SB diameter was also calculated. A value < 50% is quoted as risk factor for events in the latest update of the EAPCI bifurcation club document [14]. The “overlap lengthˮ was measured as the number of frames in which overlapping (crushed) struts were visible.

#### Quantitative coronary angiography (QCA)

QCA was analyzed in the local laboratory, which served as core laboratory for several multicentric studies. Previously described procedures and definitions were applied [17, 18]. Analysis was performed using the Cardiovascular Angiographic Analysis System (CAAS) II software version 8.2.2 (Pie Medical Imaging, Maastricht, The Netherlands) to determine reference vessel diameter (RVD), maximum and minimum lumen diameter as well as residual stenosis. Bifurcation angles were calculated with a dedicated software (CASS, Philips, The Netherlands, Version 8.5).

### Clinical endpoints

Clinical endpoints based on standardized definitions [19] included protocol success (whether the target-lesion intervention was performed according to randomization and per study protocol), device success (target-lesion stent implantation according to protocol and randomization with a final thrombolysis in myocardial infarction flow 3 and a residual stenosis < 20%), procedure success (device success and no peri-procedural complications including myocardial infarction, death, stent thrombosis, by-pass surgery, peri-procedural cardiac biomarker release according to the third universal definition of myocardial infarction at discharge), as well as peri-procedural biomarker release > 5 times upper level of normal alone. Procedural time was the interval between first LM angiography and the OCT performed at the end of the PCI. One-month clinical follow-up was performed by office visit or telephone contact. Procedural and clinical data were entered into electronic case-report forms, verified by staff not involved in other procedures, and analyzed in an anonymous way.

### Statistical analysis

Continuous variables are presented as mean ± SD or median [interquartile range] and were compared using a parametric or nonparametric tests based on the inspection of the Q–Q plots. Categorical variables are presented as counts and percentages. Since ostial SB restenosis is by far the most common mechanism of target-lesion failure after bifurcation interventions [12] and stent expansion is a major predictor of restenosis, the main endpoint of the study was ostial SB stent expansion. The power calculation was based on data from the DK-crush I trial comparing traditional crush with DK-crush [5] and reporting a 72 ± 11.5% expansion in the DK-crush and 53 ± 13% in the traditional crush group. The study was designed as a continuous outcome trial assuming a standard deviation of 11% and non-inferiority limit of 11%, i.e., 50% of the advantage of DK-crush versus traditional crush. OCT and QCA parameters were compared after final kissing balloon and before any further dilation guided by the final OCT results. Given the relatively small sample size, the Mann–Whitney *U* test was used for the analysis of continuous variables. All secondary endpoints were analyzed by descriptive statistics and appropriate exploratory *P* values.

The primary analysis was performed on a per-protocol population (i.e., all patients who are not protocol violators), a separate analysis on an intent-to-treat population (i.e., all randomized patients randomized to a treatment arm) was, however, also planned. By excluding protocol violators, the per-protocol population may be the more conservative for a non-inferiority comparison, but is susceptible to bias in either direction. A safety analysis population including all patients enrolled in the study was planned for the assessment of side effects and adverse events. A *P* value < 0.05 was considered statistically significant for the primary endpoint. Statistical analysis was performed with Medcalc (Mariakerke, BE).

## Results

Fifty-two patients with a complex left main bifurcation lesion, a Syntax score of 25 [22–29], and heart team decision to perform PCI were randomized (Supplemental Fig. 2) between 11–2018 and 11–2020 to receive either reverse-TAP (*n* = 26) or DK-crush (*n* = 26). Baseline clinical characteristics are presented in Table 2, procedural characteristics are presented in Table 3, and angiographic characteristics are presented in Table 4. There was no difference in any of the parameters between groups.

**Table 2:** Patient characteristics

	DK-crush		Reverse-TAP		*p*
	*N*, Median	IQR	*N*, Median	IQR	
Patient characteristics					
Age [years]	68	64–79	72	65–80	0.504
Female sex [*n*]	8		5		0.522
Syntax score	25	23–31	24.5	22–28	0.332
BMI [kg/m^2^]	27.0	24–29	26	25–28	0.927
Obesity [*n*]	6		5		1.000
Smoking [*n*]	1		2		1.000
Prior smoking [*n*]	11		9		0.776
Hyperlipidemia [*n*]	16		14		0.778
Hypertension [*n*]	20		25		0.104
Diabetes [*n*]	10		7		0.554
Prior CABG [*n*]	2		2		0.603
Prior PCI [*n*]	12		9		0.572
Prior stroke/TIA [*n*]	1		2		1.000
Clinical presentation					0.273
Stable angina or silent ischemia [*n*]	19		18		
Unstable angina [*n*]	3		2		
NSTEMI [*n*]	4		6		
Baseline CK [U/l]	76	46–139	75	55–108	0.892
Baseline troponin [pg/ml]	8	5–19	13	3–45	0.341
BNP [pg/ml]	92	24–153	77	40–158	0.917
Creatinine [mg/dl]	1.0	0.9–1.2	1.0	0.9–1.2	0.516
eGFR [ml/min/1.73m^2^]	78	63–93	71	64–87	0.355
LVEF [%]	55	54–60	55	50–55	0.087
Heart rate [beats/min]	70	60–75	73	63–91	0.141
Diastolic blood pressure[mmHg]	68	61–80	70	59–75	0.489
Systolic blood pressure [mmHg]	138	111–150	129	104–150	0.431
Lesion characteristics					
Medina 1.1.1 [*n*]	24		26		0.354
Medina 1.1.0 [*n*]	1		0		
Medina 0.1.1 [*n*]	1		0		
Calcification of MB [*n*]	26		24		0.471
Calcification of SB [*n*]	25		23		0.603
Lesion length > 10 mm [*n*]	20		22		0.948
Multiple bifurcations [*n*]	3		4		1.000
Main vessel < 2.5 mm [*n*]	0		4		0.119
Severe calcification [*n*]	20		16		0.367
Bifurcation angle > 70 or < 45° [*n*]	22		22		0.700

*MB* main branch, *SB* side branch, *CABG* coronary artery by-pass graft, *PCI* percutaneous coronary intervention, *TIA* transient ischemic attack, *NSTEMI* non-ST-elevation myocardial infarction

Values are presented as number (*N*) for categorical variables and median [interquartile range] for discrete or continuous variables

**Table 3:** Procedural characteristics

	DK-crush		Reverse-TAP		*p*
	*N*, Median	IQR	*N*, Median	IQR	
Procedural characteristics					
Bifurcation angle before PCI	64	50–98	75	53–100	0.443
Bifurcation angle after PCI	70	58–83	71	47–85	0.785
Total heparin, IU	7750	7500–10,000	7500	5000–10,500	0.985
Radial access [*n*]	25		26		1.000
Procedural time, min*	**32**	**24–44**	**25**	**22–33**	**0.044**
Contrast, ml	188	150–288	136	115–247	0.156
Plaque debulking [*n*]					0.334
Scoring	0		1		
Rotablation	1		3		
IIbIIIa [*n*]	4		5		1.000
Multiple PCIs [*n*]	12		10		0.779
Predilation balloon diameter MB [mm]	3	3–3	3	3–3	0.855
Predilation balloon diameter SB [mm]	3	3–3	3	2.750–3	0.956
Stent diameter MB [mm]	3.5	3–3.5	3.5	3.0–3.5	0.805
Stent diameter SB [mm]	3.0	3.0–3.5	3.0	2.75–3	0.558
Final kissing balloon diameter MB [mm]	3.0	3.0–3.0	3.0	3.0–3.0	0.634
Final kissing balloon pressure MB, atm	**14**	**12–16**	**13**	**12–14**	**0.056**
Final kissing balloon diameter SB [mm]	3.0	2.75–3.0	3.0	3.0–3.0	0.756
Final kissing balloon pressure SB, atm	**14**	**12–16**	**13**	**12–14**	**0.043**
Final POT balloon diameter [mm]	4.0	3.5–4.0	4.0	4.0–4.5	0.274
Therapy with anticoagulants [*n*]	5		4		1.000
DAPT type [*n*]					0.567
Clopidogrel	2		4		
Ticagrelor	12		13		
Prasugrel	12		9		

*p* values below 0.05 are marked in bold

*DAPT* dual antiplatelet therapy, *POT* proximal optimization technique, *MB* main branch, *SB* side branch

Values are presented as number (*N*) for categorical variables and median [interquartile range] for discrete or continuous variables

*Time from the first angiography and the protocol-mandated OCT

**Table 4:** OCT analysis

	DK-crush		Reverse-TAP		*p*
	Median	IQR	Median	IQR	
Carina–anticarina segment, OCT analysis					
Number of frames	13	10–21	13	12–18	0.82
Number of struts	172	101–224	154	124–199	0.798
Average number of struts per frame	11.6	9.3–14.1	11.1	9.5–13.5	0.552
% Struts apposed	87.5	75.6–96.7	89.1	83.6–99.5	0.245
% Malapposed (any malapposition)	12.5	3.3–24.4	10.9	0.5–16.4	0.284
% Malapposed > 250microm	6.8	1.2–13.3	8.3	1.4–15.0	0.956
Mean SB opening, %	79	71–94	88	73 – 96	0.234
Minimum SB opening, %	42	31–77	60	34–83	0.151
MB ostium expansion, %	82.0	71.0–91.7	83.7	72.4–93.6	0.540
SB ostium expansion, %	**74.6**	**66.6–89.4**	**86.1**	**75.4–95.3**	**0.037**
MB ostium eccentricity index	0.76	0.72–0.86	0.72	0.66–0.82	0.112
SB ostium eccentricity index	0.73	0.69–0.87	0.81	0.74–0.87	0.426
Overlap length	25	17–36	11	1–23	0.010

*p* values below 0.05 are marked in bold

Values are presented as number (*N*) for categorical variables and median [interquartile range] for discrete or continuous variables

Briefly, mean age was 71 [65–80]years, 17 patients had diabetes, 27 had 3-vessels disease, and 15 presented with an acute coronary syndrome at admission (NSTEMI in 10 cases, unstable angina in 5). Nine patients were on oral anticoagulants. All procedures (except for one) were performed using a radial approach, and an additional PCI was performed in 22 cases. All lesions fullfilled the DK-Crush V criteria for a complex lesion [7]. Medina class was 1,1,1 in all patients randomized to reverse-TAP and in 24 patients in the DK-crush group. Plaque debulking techniques were used in 5 patients (4 rotablation, 1 scoring balloon).

Per-protocol PCI (including POT and final kissing) was performed in all cases; therefore, the per-protocol and intention-to-treat populations overlap. Procedural characteristics were similar between groups. There was no difference in any of the lesion preparation parameters, stent size, or implantation pressure. Postdilation balloon sizes were similar, but final kissing balloon pressures were higher in the DK-crush group (main branch: DK-crush: 14 [12–16] ATM vs reverse-TAP: 13 [12–14] ATM, *P* = 0.056; side branch: 14 [12–16] vs 13 [12–14] ATM respectively, *P* = 0.043). Procedural time was higher in the DK-crush group (32 [24–44] min vs 25 [23–33] min, *P* = 0.044). The use of contrast was not different between groups, but it showed a trend toward less injected volume in the reverse-TAP group (188 [150–288] ml vs 136 [115–247] ml, *p* = 0.156).

### Procedural and 1-month outcomes

The interventions were performed per-protocol in all patients (*P* = 1.000). Device success was achieved in all but 2 in the DK-crush and 2 in the reverse-TAP group (*P* = 1.000, residual QCA stenosis > 20% and < 25% in all cases). Additionally, there was 1 intraprocedural in-stent thrombosis in a non-target lesion in the DK-crush group (procedural success *P* = 1.00). Despite per-protocol PCI, the OCT catheter could not cross the bifurcation into the side branch in two cases, both in the DK-crush group. Final OCTs of the SB were therefore available in, respectively, 24 (DK-crush) and 26 (reverse-TAP) patients. QCA data were available for all patients. After OCT, an additional (non-protocol driven) balloon dilation was performed in 12 patients (5 in the DK-crush and 7 in the reverse-TAP group, *P* = 0.742). During 1-month follow-up, no events were recorded.

### Primary endpoint

**Fig. 2:**
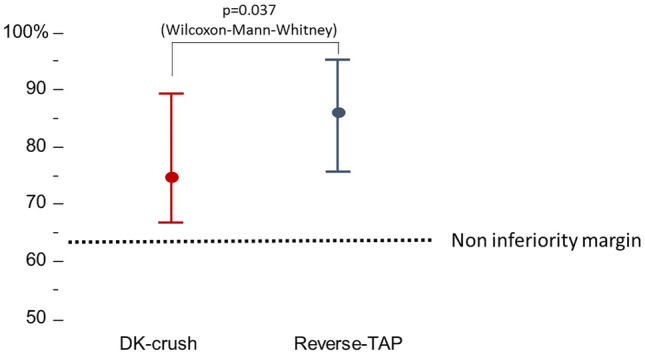
Primary endpoint assessment in the full analysis set population (data are presented at median [IQR] due to non-normal distribution). The lower confidence interval of the differences was higher than the pre-specified non-inferiority margin of 11%. In addition, SB expansion achieved with reverse-TAP was larger than with DK-crush by Mann–Whitney *U*

As measured by OCT, the final post-PCI ostial side branch expansion (primary outcome) was 74.6 [66.6–89.4]% in the DK-crush group and 86.1 [75.4–95.3]% in the reverse-TAP group (Table 4). Ostial side branch expansion was non-inferior (one-sided 97.5% lower parametric CI − 0.28%; non-inferiority *p* < 0.01) and superior (*p* = 0.037 by Wilcoxon–Mann–Whitney, Fig. 2) in the reverse-TAP group as compared to the DK-crush. The SB minimum diameter at the ostium was in all patients > 50% the reference SB diameter, and a comparison between groups showed a larger ratio in the reverse-TAP group (87 [79–92% vs 92 [86–96]%, *p* = 0.032).

### QCA analysis

Data are presented in Supplemental Table 1. There was no difference between groups in any of the variables before PCI or after PCI. The luminal gain was also not different (proximal main: *P* = 0.971; MB: *P* = 0.608; SB: *P* = 0.993). The stent/artery ratio was 1.1 [1.1–1.2] without between-group difference in the main branch and 1.1 [1.0–1.1] in the side branch in DK-Crush as opposed to 1.1 [1.1–1.2] in the reverse-TAP group. The angle between SB and MB did not differ between groups.

### Other OCT endpoints

Data are presented in Table 4 and supplemental Table 2. No gap was found at the level of the SB ostium and in the carina–anticarina segment in either group. Overall, 11.8 [2.8–17.1]% of the struts in the carina–anticarina segment were malapposed (*p* = 0.284 between groups); 6.8 [1.2–13.3]% (DK-crush) and 8.3 [1.4–15.0]% (reverse-TAP) of the struts were malapposed more than 250 µm (*P* = 0.956). The overlap length was significantly shorter in the reverse-TAP group (Table 4, *P* = 0.010).

The minimum and mean side branch opening were, respectively, 50 [34–80]% (*p* = 0.151) and 83.0 [72–95]% (*p* = 0.234), without differences between groups. The stent eccentricity index at the ostium of the MB and the SB was, respectively, 0.76 [0.69–0.85] (*p* = 0.112 between groups) and 0.79 [0.70–0.80]% (*p* = 0.426 between groups) (Table 5).

**Table 5:** Clinical outcomes

	DK-crush		Reverse-TAP		*p*
	*N*, Median	IQR	*N*, Median	IQR	
Procedural outcomes					
Protocol deviation [*n* of patients]	0		0		1.000
Device success [*n*]	24		24		1.000
Procedural success [*n*]	23		24		1.00
Troponin elevation > 5 times ULN [*n*]	13		16		1.000
Peak troponin [ng/ml]	206	64–1431	289	31–558	0.672
Post-OCT optimization [*n*]	5		7		0.742

*ULN* upper level of normal

Values are presented as number (*N*) for categorical variables and median [interquartile range] for discrete or continuous variables

## Discussion

Coronary bifurcations represent a challenging subset of lesions for percutaneous coronary interventions, in which the technique used is an important predictor of patient outcomes. Provisional stenting—with or without final kissing with a standard or drug-eluting balloon—is the simplest technique for bifurcations, and recent data appear to support this approach [2]. Two-stent techniques are more complex, and the literature offers a wide range of theoretical options as summarized by the Main, Across, Distal, Side (MADS) classification (latest updated in [4]). Of these, the one with the most promising results appears to be the so-called Double-Kissing (DK) crush technique, which consists of several steps (Table 1 and [20, 21]), with numerous variations and additions since 2005. In the DK-crush V trial, compared with PS, DK-crush was superior in terms of target-lesion failure, target vessel myocardial infarction, and definite or probable stent thrombosis [7].

In the MADS classification, the acronym “TAP” is currently used to describe a variation of provisional stenting (“classical TAP”) or for a 2-stent technique in which the second stent is implanted in the ostial MB with protrusion (“inverted TAP”) [4]. Based on the MADS classification nomenclature, the strategy used in the present study would be described as “side branch first with TAP, high-pressure side, and alternate dilations, MB stent, followed by POT-Kissing-POT”. This technique differs from DK-crush in several details (Table 1): first, it foresees a systematic repeated high-pressure dilation of the SB stent at the ostium, which might explain the larger ostial SB stent expansion. Second, since the goal of the sequential balloon inflations is to distribute the SB stent struts along the circumference of the SB ostium, the protrusion of the SB stent in the MB is smaller in reverse-TAP, as confirmed by the shorter “overlap length”. Third, while for DK-crush, both rewiring are performed through a side cell of the SB stent, in the reverse-TAP rewiring is planned to occur through the lumen of the SB stent. The goal of the repeated PTCA after SB stent implantation is to optimize the opening of the SB stent ostium to facilitate rewiring. This may have the advantage of limiting the amount of overlapping metal in the mother vessel, a potential source of late failure. As well, the absence of complete crushing allows continuing the procedure without having to perform the first rewiring. The SB wire can be maintained in place during MB stenting. This improves the chance of patency of the side branch and provides a marker facilitating rewiring. Collectively, these small differences might explain the shorter procedural time reported in the present study with reverse-TAP as compared to DK-crush. All QCA and OCT parameters, along with procedural outcomes, appear to support the equivalence of the two techniques.

### Limitations

This was a small trial with an imaging endpoint and any conclusion about hard outcomes is purely speculative. We do not believe that one single-stent strategy may be applied to all anatomical settings. A radical simplification of the bifurcation treatment (provisional stenting, including potentially drug-coated balloon PTCA in the non-stented side branch ostium) might also be a valid alternative in many, if not most, settings. Also, specific subsets (e.g., trifurcation lesions) were excluded. The study was conducted in only one center by operators experienced with both techniques to limit inter-operator variability. In the protocol used for DK-crush, proximal optimization was not performed before implantation of the MB stent. This additional step has been officially introduced only in 2020, and it might improve the outcome of 2-stent PCIs (both reverse-TAP and DK-crush). Furthermore, OCT was performed only at the end of the PCI. The importance of OCT before or during PCI cannot be emphasized enough. However, in a trial focusing on outcomes of stenting strategies, additional OCT runs would have introduced variations in the technique deviating from the intended protocol and not allowing a direct comparison between strategies. OCT was performed after completing the study protocol to collect the primary endpoint and guide (when necessary) further clinically indicated optimization. Xience (Abbott Vascular, Santa Clara, USA) stents were used for all patients. We cannot exclude that the results might have been different with other stent types. The originally proposed protocol for DK-crush was used in this trial. Changes that have been added during the course of the years (e.g., dilation with a larger NC balloon at the ostium of the SB immediately following the SB stenting) might improve the results of this strategy. Finally, the study was conducted in LM bifurcations, and very narrow bifurcations, particularly in smaller vessels, represent an additional challenge. A narrower bifurcation angle is a challenge which increases the risk of gaps between the two stents. These lesions are probably better treated with DK-crush.

## Conclusions

In a randomized, controlled procedural outcome trial comparing two stenting strategies for the treatment of complex unprotected left main bifurcation stenoses, reverse-TAP proved non-inferior and superior to DK-crush while also reducing procedural time. Of note, the improvement (or non-inferiority) of an OCT endpoint does not allow any conclusions regarding the clinical implications of different stenting strategies. Future larger, multicentric studies will have to compare the clinical outcomes of the two techniques.

## Supplementary Information

Below is the link to the electronic supplementary material.

Supplementary file1 (DOCX 619 KB)

## Abbreviations

ACS: Acute coronary syndromes
CI: Confidence interval
DES: Drug eluting stents
DK-crush: Double kissing and crush
MLD and RVD: Minimum lumen and reference vessel diameter
(N)STEMI: (Non-) ST-elevation myocardial infarction
MB: Main branch
OCT: Optical coherence tomography
PCI: Percutaneous coronary intervention
POT: Proximal optimization technique
SB: Side branch
SD: Standard deviation
TAP: T-and-protrusion
TLR: Target lesion revascularization
TLF: Target lesion failure
QCA: Quantitative coronary angiography
