# Supplementary material for: Randomized non-inferiority TrIal comParing reverse T And Protrusion versus double-kissing and crush Stenting for the treatment of complex left main bifurcation lesions
Source: Clin Res Cardiol. 2021 Nov 24;111(7):750–60. doi: 10.1007/s00392-021-01972-2 (PMC9242916; doi:10.1007/s00392-021-01972-2)

**Randomized, non-inferiority, TrIal comParing reverse T And Protrusion versus double-kissing and crush Stenting for the treatment of complex left main bifurcation lesions.**

**Supplemental data**

Maximilian Olschewski (maximilian.olschewski@unimedizin-mainz.de); Helen Ullrich ([Helen.ullrich@unimedizin-mainz.de](mailto:Helen.ullrich@unimeidzin-mainz.de)); Maike Knorr ([maike.knorr@unimedizin-mainz.de](mailto:maike.knorr@unimedizin-mainz.de)); Majid Ahoopai ([majid.ahoopai@unimedizin-mainz.de](mailto:majid.ahoopai@unimedizin-mainz.de)); Thomas Münzel ([tmuenzel@uni-mainz.de](mailto:tmuenzel@uni-mainz.de)); Tommaso Gori ([tommaso.gori@unimedizin-mainz.de](mailto:tommaso.gori@unimedizin-mainz.de))

From the Department of Cardiology, University Medical Center Mainz, Mainz, Germany and German Center for Cardiac and Vascular Research (DZHK), Standort Rhein-Main

Address for correspondence:

Tommaso Gori

Zentrum für Kardiologie

University Medical Center Mainz

Langenbeckstrasse 1

55131 Mainz

Tel: +49 6131 17 2829

Fax.: +49 6131 17 6428

Email: tommaso.gori@unimedizin-mainz.de

**Inclusion and exclusion criteria:**

Inclusion criteria were a presentation with silent ischemia, stable or unstable angina, or myocardial infarction >24 hours, a true bifurcation stenosis (Medina type 1,1,1 or 0,1,1) with branches >2.5 and <5mm with angiographic stenosis >50%, positive non-invasive imaging for ischemia compatible with LM stenosis OR positive Fractional Flow Reserve OR mean lumen area (MLA) <6mm^2^ for the left main or <4mm^2^ for ostial epicardial vessels as assessed by intracoronary imaging, age ≥18 years. Exclusion criteria were cardiogenic shock, trifurcation lesions, either bifurcation vessel not suitable for stenting, history of stenting in target lesions, inability to give informed consent, child-bearing potential or lactation.

**Main differences between reverse-TAP and similar stenting techniques**

The „reverse-TAP“ technique employed in the current trial differs slightly from previously proposed two-stent techniques:

- „Nano-crush“ (PD Morris et al. Can J Cardiol, 36, 2020, 852-859): this technique is very similar to the cone flare crush (see below). The main difference with reverse TAP is that the initial kissing of nano-crush (performed with the stent balloon in the SB and the sentinel balloon in the MB) is aimed at creating a neocarina (from the paper by Morris et al: „The purpose of this manoeuvre is to ensure that there is sufficient space to advance the MB stent while keeping the proximal lateral border of the SB stent well opposed at the bifurcation“).

These struts jailed between the two kissing balloons will form a carina which remains until the end of the procedure (red arrow, figure from PD Morris et al. Can J Cardiol, 36, 2020, 852-859):


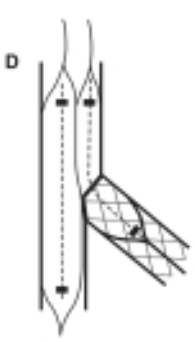

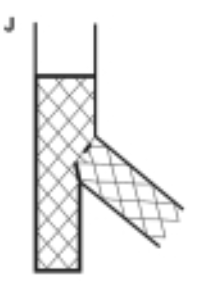


In the reverse TAP method presented here, goal of the sequential kissing (as in the standard TAP technique described in the 13th EBC consensus document, page 118) is to improve MB and SB ostium expansion while also shifting the neocarina forward, thus minimizing its longitudinal extent (the white arrow describes the direction towards which the struts are pushed; by repeating this process the intent is that the carina can be moved forward).


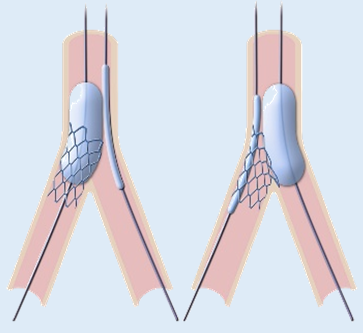


- „Modified T stent“ (Iakovou 2005): „this Modified T stenting is a variation performed by simultaneously positioning stents at the SB and the MB. The SB stent is deployed first, and then after wire and balloon removal from the SB, the MB stent is deployed.“. In the reverse-TAP used here, stents are placed sequentially, and there is a kissing PTCA before the main vessel stent is positioned.

- „Cone Flare Crush Modified-T stenting technique“ (S. Rajdev et al, ‘‘Cone Crush”, a variant of modified T-Stenting technique for coronary bifurcation lesions: bench testing, clinical feasibility, and in-hospital outcomes, J. Am. Coll. Cardiol. 49 (S9) (2007) and Peverill WT et al. Int J Cardiol Heart Vasc. 2020 Sep 25;30:100643): this technique, first described in a case report and then in a single-arm case series of 20 patients, is very similar to the reverse TAP used here. The main difference includes the sequential high-pressure dilation prior to the first kissing PTCA (Table 1, row 4 in the reverse-TAP column). This sequential PTCA is recommended in the „standard“ TAP method as described in the EBC white paper: „Perform kissing balloon inflation using short balloons and MV balloon sized 1:1 according to the distal MV diameter. According to bench test data, minimal balloon overlap in the proximal MV, with sequential balloon inflation (alternate inflation of MV and SB balloon followed by simultaneous inflation/deflation) using non-compliant balloons is advisable in order to minimize stent distortion and SB dissection risk“ (Burzotta F, „European Bifurcation Club white paper on stenting techniques for patients with bifurcated coronary artery lesions“, CCI 2020).

Supplemental Table 1. Quantitative coronary analysis data

|  |  | | | |  |
| --- | --- | --- | --- | --- | --- |
|  | DK Crush | | Reverse TAP | |  |
|  | Median | IQR | Median | IQR | p |
| Mother vessel |  |  |  |  |  |
| Minimum lumen diameter before PCI, mm | 2.4 | 2.2-2.8 | 2.0 | 1.7-2.4 | 0.091 |
| Minimum lumen diameter after PCI, mm | 3.6 | 3.2-4.0 | 3.4 | 3.0-3.6 | 0.118 |
| Reference vessel diameter, mm | 3.7 | 3.5-4.0 | 3.6 | 3.3-3.9 | 0.440 |
|  |  |  |  |  |  |
|  |  |  |  |  |  |
| Main vessel |  |  |  |  |  |
| Minimum lumen diameter before PCI, mm | 1.8 | 1.4-2.1 | 1.8 | 1.7-2.7 | 0.935 |
| Minimum lumen diameter after PCI, mm | 2.8 | 2.5-3.0 | 2.7 | 2.5-3.0 | 0.583 |
| Reference vessel diameter, mm | 3.0 | 2.7-3.3 | 3.0 | 2.9-3.2 | 0.927 |
|  |  |  |  |  |  |
|  |  |  |  |  |  |
| Side vessel |  |  |  |  |  |
| Minimum lumen diameter before PCI, mm | 1.7 | 1.4-2.0 | 1.5 | 1.1-1.8 | 0.123 |
| Minimum lumen diameter after PCI, mm | 2.6 | 2.3-2.8 | 2.5 | 2.2-2.6 | 0.096 |
| Reference vessel diameter, mm | 2.8 | 2.5-3.1 | 2.7 | 2.4-2.9 | 0.182 |
|  |  |  |  |  |  |
|  |  |  |  |  |  |
| Residual stenosis, % |  |  |  |  |  |
| Mother vessel | 4.9 | 0.6-9.0 | 5.9 | 2.7-10.7 | 0.346 |
| Main vessel | 6.6 | -0.4-14.9 | 6.4 | 3.6-13.4 | 0.905 |
| Side vessel | 7.8 | 2.4-10.4 | 9.5 | 1.4-14.1 | 0.708 |

Supplemental Table 2. Optical coherence tomography data

|  |  | | | |  | | |
| --- | --- | --- | --- | --- | --- | --- | --- |
|  | DK Crush | | Reverse TAP | |  | | |
|  | Median | IQR | Median | IQR | p | | |
| Mother vessel |  |  |  |  |  | |  |
| max. stent area  [mm²] | 11.3 | 10.2-13.1 | 12.6 | 10.6-15.5 | 0.228 | |  |
| max. stent diameter  [mm] | 3.8 | 3.6-4.1 | 4.0 | 3.7-4.5 | 0.132 | |  |
| mean stent area  [mm²] | 9.7 | 9.0-11.0 | 10.2 | 8.8-12.2 | 0.578 | |  |
| mean stent diameter  [mm] | 3.6 | 3.4-3.7 | 3.7 | 3.4-3.9 | 0.412 | |  |
| min. stent area  [mm²] | 8.5 | 7.3-9.7 | 8.3 | 6.2-10.6 | 0.924 | |  |
| min. stent diameter  [mm] | 3.3 | 3.1-3.5 | 3.3 | 2.8-3.7 | 0.770 | |  |
| average stent eccentricity index, % | 88 | 80-92 | 85 | 73-88 | 0.132 | |  |
|  |  |  |  |  |  | |  |
| Main branch |  |  |  |  |  | |  |
| max. stent area  [mm²] | 8.5 | 7.8-9.5 | 8.8 | 7.6 -10.3 | 0.720 | |  |
| max. stent diameter  [mm] | 3.3 | 3.1-3.5 | 3.3 | 3.1-3.6 | 0.735 | |  |
| mean stent area  [mm²] | 7.2 | 6.3-8.3 | 7.6 | 6.0-8.8 | 0.578 | |  |
| mean stent diameter  [mm] | 3. | 2.8-3.2 | 3.1 | 2.7-3.3 | 0.578 | |  |
| min. stent area  [mm²] | 5.7 | 4.9-6.9 | 6.3 | 4.9-6.9 | 0.572 | |  |
| min. stent diameter  [mm] | 2.7 | 2.5 – 3.0 | 2.8 | 2.5-3.0 | 0.572 | |  |
| average stent eccentricity index, % | 83 | 75-88 | 85 | 78-88 | 0.313 | |  |
|  |  |  |  |  |  | |  |
| Side branch |  |  |  |  |  | |  |
| max. stent area  [mm²] | 8.2 | 7.0-9.7 | 7.6 | 5.9- 8.5 | 0.214 | |  |
| max. stent diameter  [mm] | 3.2 | 3.0-3.5 | 3.1 | 2.7-3.3 | 0.197 | |  |
| mean stent area  [mm²] | 6.8 | 5.8-8.3 | 6.8 | 5.4-7.5 | 0.378 | |  |
| mean stent diameter  [mm] | 2.9 | 2.7-3.2 | 3.0 | 2.6-3.2 | 0.642 | |  |
| min. stent area  [mm²] | 5.3 | 4.4-6.4 | 5.3 | 4.2-6.6 | 0.846 | |  |
| min. stent diameter  [mm] | 2.6 | 2.3-2.9 | 2.6 | 2.3-2.9 | 0.985 | |  |
| average stent eccentricity index, % | 81 | 76-88 | 87 | 80-90 | 0.134 | |  |
|  |  |  |  |  |  | |  |
| MB ostium area[mm]² | 7.2 | 5.4-8.2 | 7.3 | 6.2-8.5 | 0.685 |  |  |
| MB ostium average diameter[mm] | 3.0 | 2.6-3.2 | 3.0 | 2.8-3.3 | 0.699 |  |  |
| MB ostium maximum diameter[mm] | 3.3 | 3.0-3.6 | 3.4 | 3.1-3.9 | 0.113 |  |  |
| MB ostium minimum diameter[mm] | 2.7 | 2.3-2.9 | 2.6 | 2.2-2.9 | 0.658 |  |  |
| SB ostium area[mm]² | 5.7 | 5.2-7.0 | 5.8 | 4.80-7.7 | 0.977 |  |  |
| SB ostium maximum diameter[mm] | 3.2 | 2.8-3.4 | 3.0 | 2.7-3.6 | 0.573 |  |  |
| SB ostium minimum diameter[mm] | 2.3 | 2.1-2.7 | 2.4 | 2.1-2.7 | 0.756 |  |  |
| SB ostium average diameter[mm] | 2.7 | 2.6-3.0 | 2.7 | 2.5-3.1 | 0.992 |  |  |
|  |  |  |  |  |  | |  |

Supplemental Figure 1

Side branch opening was calculated according to (12) as mean and minimum % ratio between the maximum strut-free distance (d) and SB ostium diameter. Measurements were performed on a frame-by-frame basis between the carina and the anti-carina. MB: main branch; SB: side branch


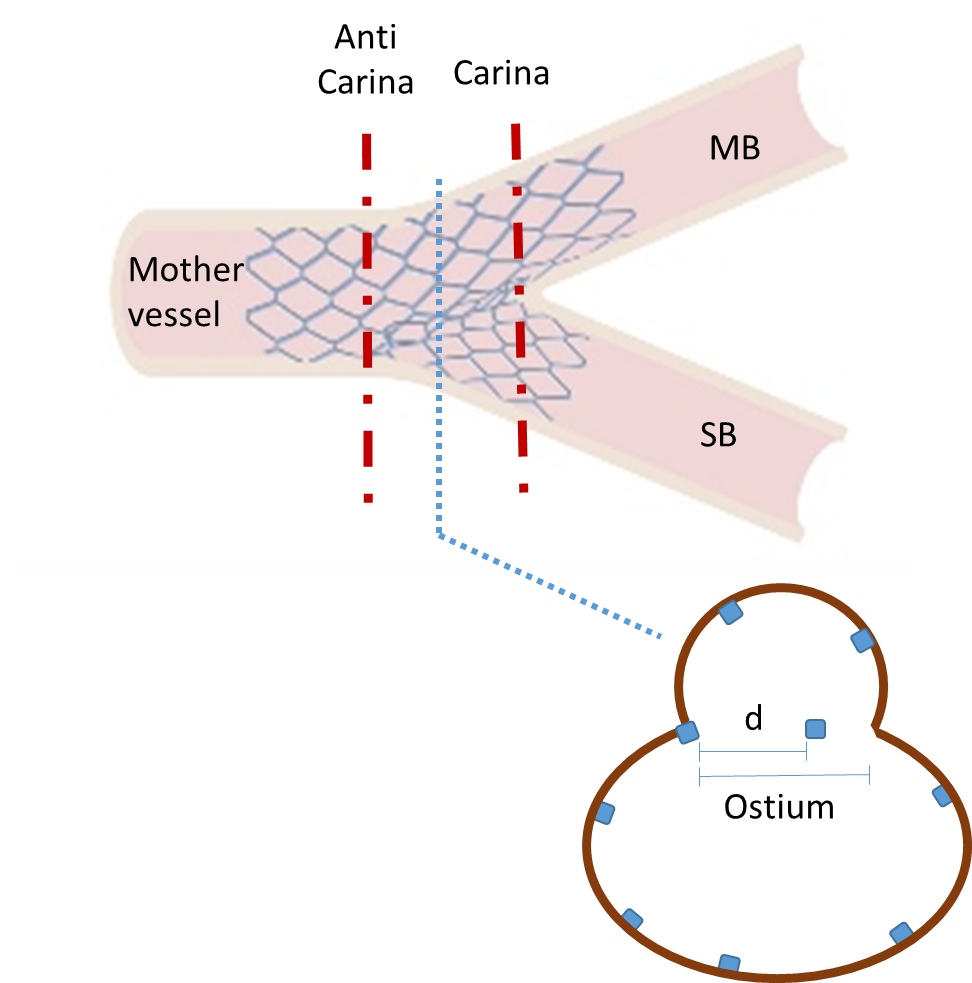


Supplemental Figure 2

Workflow of the study.


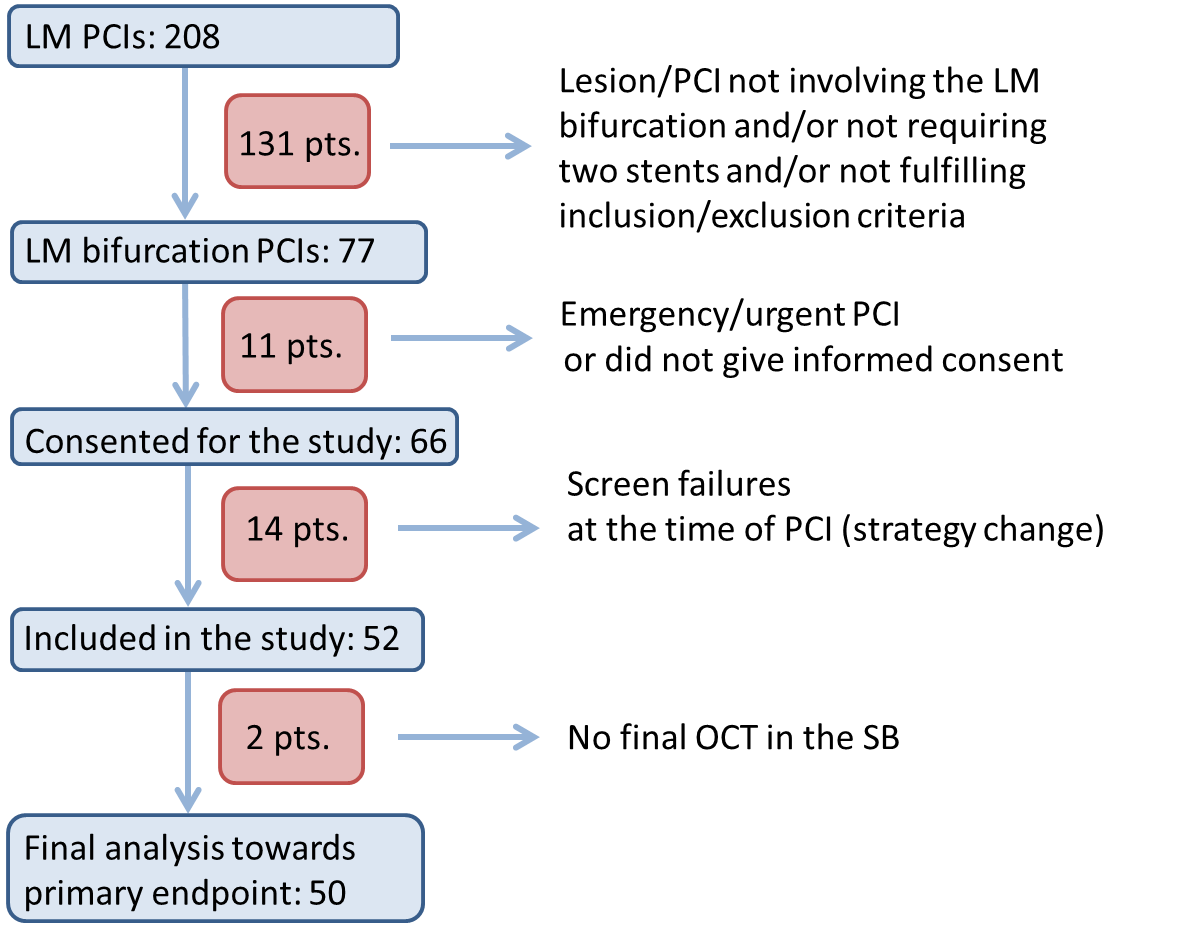

Supplement: Supplementary file 1 — Supplementary file1 (DOCX 619 KB) [file 392_2021_1972_MOESM1_ESM.docx]
